# Three-Dimensional Electrodes for High-Performance Bioelectrochemical Systems

**DOI:** 10.3390/ijms18010090

**Published:** 2017-01-04

**Authors:** Yang-Yang Yu, Dan-Dan Zhai, Rong-Wei Si, Jian-Zhong Sun, Xiang Liu, Yang-Chun Yong

**Affiliations:** Biofuels Institute, School of Environment and Safety Engineering, Jiangsu University, 301 Xuefu Road, Zhenjiang 212013, China; yyyu@ujs.edu.cn (Y.-Y.Y.); daneszh@126.com (D.-D.Z.); srw568@163.com (R.-W.S.); jzsun1002@ujs.edu.cn (J.-Z.S.); liuxiang0222@126.com (X.L.)

**Keywords:** bioelectrochemical systems, three-dimensional electrode, macroporous, nanostructure, microbial fuel cells

## Abstract

Bioelectrochemical systems (BES) are groups of bioelectrochemical technologies and platforms that could facilitate versatile environmental and biological applications. The performance of BES is mainly determined by the key process of electron transfer at the bacteria and electrode interface, which is known as extracellular electron transfer (EET). Thus, developing novel electrodes to encourage bacteria attachment and enhance EET efficiency is of great significance. Recently, three-dimensional (3D) electrodes, which provide large specific area for bacteria attachment and macroporous structures for substrate diffusion, have emerged as a promising electrode for high-performance BES. Herein, a comprehensive review of versatile methodology developed for 3D electrode fabrication is presented. This review article is organized based on the categorization of 3D electrode fabrication strategy and BES performance comparison. In particular, the advantages and shortcomings of these 3D electrodes are presented and their future development is discussed.

## 1. Introduction

The phenomenon that microorganisms could degrade substrates and transfer electrons from their central metabolism to the electrode was found more than a century ago [1]. However, it was until most recently, with the increasing concern for petroleum exhaustion and environmental pollution, that the motivation for substitutive, clean, and recyclable energy began driving research attention back to this interesting microbial physiological process. As a result, a series of biotechnologies based on microbial electroactivity were developed and the corresponding devices are generally nominated as bioelectrochemical systems (BES) [2,3,4].

The microbial fuel cell (MFC) was the first developed BES. The development of the microbial fuel cell was inspired by the following contradictory facts: treatment of domestic wastewater via conventional aerobic treatment costs 0.5 kWh per m^3^ of electrical power; meanwhile the energy dissipation within the biomass oxidation is 2.21 kWh per m^3^, around 4.4 times the electrical power consumed [5]. Thus, developing suitable techniques which can recover the energy in wastewater would effectively compensate the energy consumption during the wastewater treatment and even achieve a net energy output. Compared with conventional techniques for biomass energy harvest, like anaerobic digestion, the microbial fuel cell is the most promising biotechnology for recovering energy from wastewater as it enables the treatment of the wastewater with low biomass content at room temperature [5,6,7].

Although the MFC power output capability has been improved a thousand fold during the past decade, direct use of MFC for electricity generation is still difficult. Instead, researchers developed more versatile bioelectrochemical systems, in which both anode and cathode reactions can be used to fulfill identical bioelectrochemical transformation. The representative applications include contaminant degradation and bioremediation [8,9,10,11,12], MFC-assisted desalination [13,14,15,16,17], bioproduction of recyclable energy and valuable chemicals [18,19,20,21,22,23,24,25,26,27], and biosensoring [28,29,30,31,32,33,34,35,36,37].

Despite the various potential applications of BES, the key step is the extracellular electron transfer (EET) between bacteria and the electrode interface. It induces an oxidative (anode) and reductive (cathode) current flow to drive correlated biochemical reactions at the bacteria/electrode interface and electron carrier transportation both in the external and internal circuit. The distinguished pathways developed by electroactive bacteria to accomplish EET are generally categorized into two groups: direct contact-based electron transfer (DET) via cell membrane-associated compounds (outer-membrane (OM) cytochrome and conductive pili) and mediated electron transfer (MET) via soluble electron shuttle.

Within the identical BES, the current flow may be restricted by the slow electron exchange rate between the OM cytochrome and electrode [38,39], low biofilm mass amount [40], and biofilm conductivity [41], limited electron shuttle [42,43], and substrate and buffer concentration [44]. As a result, the limited EET efficiency is usually the major bottleneck of BES performance. Thus, developing new strategies and techniques to improve the EET efficiency is of great significance and attracts vast research interest. Among the extensive successful attempts, design and fabrication of novel electrode material is the most impressive achievement during the past decades.

Compared with planktonic cells, the EET of microorganisms directly attach or agglomerate on the electrode surface (known as biofilm) are more efficient since it is spatially favorable. The dominant role of electrode-attached bacteria in EET gives rise to the specific requirement for BES electrode material. In addition to the general characteristics of electrode material, such as good conductivity, excellent longevity, and cost-effectiveness, additional requirements should be considered, which include good biocompatibility, large surface area, and suitable surface properties for bacteria attachment and electron transfer [45]. Furthermore, the diffusion at the biofilm/electrode interface also needs to be considered [46] Taking into account all of the above specific requirements, carbon-based materials are the major choice for lab-sized BES application.

Diverse kinds of carbon materials have been applied for BES studying, which cover a long list including glassy carbon, carbon paper, carbon cloth, carbon felt, graphite plate, granule graphite, granule active carbon, carbon mesh, reticulated vitrified carbon, and graphite brush [47,48]. Most carbon materials, such as glassy carbon, carbon paper, carbon cloth, are composed of plane 2D structures with identical surface morphology and property. The electrochemical property of these pristine carbon materials can be effectively improved by proper surface treatment (acid, heat, amino gas, etc.) [49,50] and modification (nanomaterials, conductive polymer, and immobilized electron shuttle) [38,51,52,53], resulting in significantly improved BES performance. However, the planar structure of these carbon materials provides limits surface area for electroactive bacteria attachment and restricts efficient substrate and buffer diffusion. Further improvement is difficult and the electrode structure must be redesigned.

Different from the 2D electrode, a three-dimensional electrode with open macroporous structure provides a large surface area for bacteria attachment and enables the formation of 3D biofilm [54]. Inspired by their distinguished advantages, 3D electrode materials attracted great attention for BES application over the last decade and impressive progress has been made. In the following part of this review article, we will mainly focus on the versatile strategies developed for 3D electrode design and the dialectical comparison of BES performance with different 3D electrode configurations. Both advantages and disadvantages will be considered for each 3D electrode developing strategy. We hope this review article will provide a good summary for the scattered attempts contributed by the researchers around the world and be a meaningful reference for those who decide to continue the work in related areas.

## 2. Building 3D Electrodes for High-Performance BES

It is generally accepted that 3D electrodes outperform 2D electrodes in BES. However, regardless of its well-recognized physical and geometrical meaning, the concept of the as-claimed “3D electrode” is confusing if a comparative study of the related works is conducted. Due to the 3D nature of packed bed carbon granules and brush electrodes, they were the first developed 3D electrodes for BES. In packed bed MFC, granule or porous carbon was filled into the electrode chamber to allow random contact to form a porous 3D electrode with an interspace left for electrolyte flow and substrate diffusion [55,56,57]. Meanwhile, the brush structure, which consists of metal wires twisted together with clamped conducting fibers, is another kind of conventional 3D electrode. “3D” in these conventional attempts can be viewed as “occupation of the reactor chamber from three dimensions”.

Inspired by these works, versatile and fancy 3D electrodes have been demonstrated by embracing the advances in material science and nanotechnology. Compared with conventional ones, the later developed 3D electrodes put more emphases on its fine micro- and macrostructure. In some attempts, “3D electrode” referred to the nanostructure (like nanowire and nanosheet) formed on a planar surface [58]. Meanwhile, in other works, “3D electrode” means the electrode with an open macroporous structure that could facilitate the interior biofilm formation in the electrode matrix. Since the electroactive biofilm, which consists of micrometer-sized bacteria and their extracellular polymeric substances, plays essential role in BES for substrate transformation and extracellular electron transfer, 3D electrodes with μm–mm size fine structure, which is beneficial for 3D biofilm formation, has more application significance.

For systematically comparing various strategies developed for 3D electrode construction, we classified the methods reported by the literature into four groups: packed bed and brush electrodes (conventional 3D electrode); 3D matrix fabricated on 2D electrodes; monolithic 3D electrodes from 3D templates; and 3D bioelectrodes with hybridized biofilms. This article aims to provide a technical review and comparative discussion on the versatile strategies developed for 3D electrode preparation in BES studies.

Table 1 summarizes the selected literature works using 3D electrode materials for BES applications. Current density and power density normalized to electrode projected area, electrode and anode volume were adopted for performance comparison. The projected area normalized current and power density are good indicators for studying the role of “3D structure” since commercial planar electrodes were usually used as controls in these works. Power density normalized to electrode volume can be viewed as the theoretical maximum power density since, in principle, the 3D electrode can fully occupy the BES chamber. Power density normalized to chamber volume helps us to make a more reasonable comparison among different BES configurations as the increase in the chamber/electrode volume ratio would improve the electrode volume normalized power density, but reduce chamber volume normalized power density [59].

### 2.1. Conventional 3D Electrodes: Packed Bed and Brush Electrode

The development of packed bed reactors for anaerobic digestion inspired the construction of packed bed MFC, in which granule or porous carbon were filled into the MFC electrode chamber to form porous 3D electrodes with interspace left for electrolyte flow and substrate diffusion [55,56,57]. Compared with those planar electrode equipped MFCs, these packed bed MFCs had higher reactor space utilization and larger electrode surface area. As a result, the bioelectricity generation in these packed bed MFCs were improved. The maximum power density ascribed to the projected area (P^a^, as summarized in Table 1, the projected area of the separator was adopted for packed bed MFC) were reported to be 0.1–1.0 W/m^2^. Meanwhile the improved reactor space utilization greatly enhanced the reactor volumetric power density (P^b^ in Table 1) to 10–100 W/m^3^ [55,56,57,62,63,65].

The granule size has to narrow down from millimeters to micrometers for further improvement of the specific area of packed bed MFC. Meanwhile, the reduced granule size increased ohmic resistance and reduced porosity [60]. Instead, brush structure, which consists of metal wires twisted together with clamped conducting fibers, can use the micrometer-sized carbon fiber with macroporous sturcutre remaining. Logan et al. made a pioneering contribution by fabricating the brush electrode with titanium wires and graphite fibers (7.2 μm in diameter). The small fiber size led to a large specific area (18,200 m^2^/m^3^, brush with a 2.5 cm diameter and a 2.5 cm length) and high porosity (95%). The maximum power density of 2.4 W/m^2^ (normalized to cathode projected area) was achieved when this brush electrodes were used as MFC anodes, which was the highest when the work was published [116]. A successful pilot-scale MFC (90 L) with brush anodes was demonstrated and net electricity output was demonstrated [117].

### 2.2. 3D Matrix Fabricated on a 2D Electrode

Packed bed and brush electrodes belong to the conventional attempts of constructing 3D electrodes for BES application. Meanwhile, further development is difficult. Since the electroactive bacteria are micrometer-sized and the EET between bacteria and the electrode takes place at the nanometer level, elaborate strategies to fabricate 3D electrodes both with macroporous structures for biofilm formation and nanostructures for EET are of great value. Versatile attempts have been demonstrated and modifying 3D structures on 2D electrodes has attracted vast research interest with simple preparation procedures and effectiveness in improving BES performance.

#### 2.2.1. Physical Deposition and Self-Assembly

**Figure 1:**
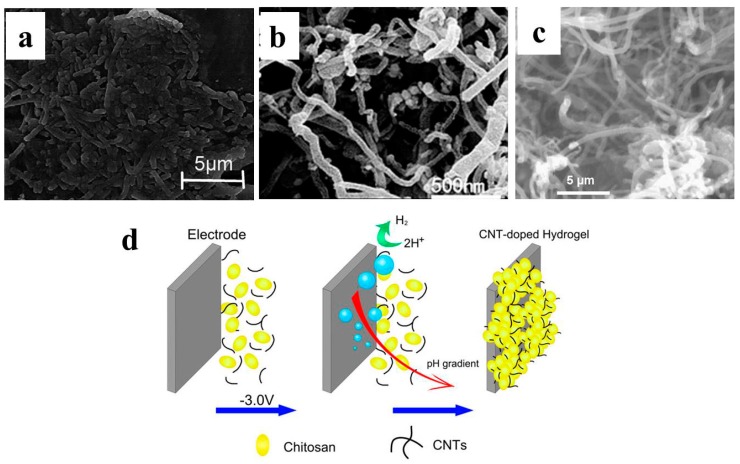
SEM images of 3D electrodes self-assembled on plane electrodes. (**a**) Biofilm formed on rGO-SnO_2_ composite on carbon cloth [69]; (**b**) plane graphite with MWCNT/MnO_2_ composite [70]; (**c**) carbon paper modified with MWCNT/Pt [71]; and (**d**) CNT/chitosan hydrogel assembled on carbon paper via electrodeposition [75]. Reproduced with permission from Elsevier and American Chemical Society.

By simply dropping or spreading the nanomaterials onto the plane electrode surface, the electrode surface property was changed and might be favorable for bacteria attachment and EET [118,119]. More importantly, 3D structure may be formed on the plane surface due to the nanomaterial’s self-assembly [74]. Mehdinia et al. successfully fabricated microwave-assisted rGO/SnO_2_ nanocomposite and carbon cloth was then modified by this composite through repeated dip and press. The 3D structure assembled on the carbon cloth was confirmed from SEM imaging (Figure 1a). With *Escherichia coli* adopted as the anode inoculum, a maximum power density of 1.62 W/m^2^ was achieved, which was almost five times that of bare carbon cloth [69]. Fu et al. synthesized MWCNT/MnO_2_ by KMnO_4_ chemical reduction. The composite powder was then pasted on planar graphite to form a porous 3D structure (Figure 1b). The maximum power density was 0.11 W/m^2^ when the modified electrode was used in marine benthic MFC, which was 10 times that of planar graphite [70]. MWCNT/Pt composite-modified carbon paper and with a 3D structure can also be formed in similar way, and significant MFC performance improvement was confirmed [71]. Liu et al. developed a self-assembled CNT/chitosan scaffold on the carbon paper electrode via electrodeposition (Figure 1d). This biocompatible composite electrode was applied both for MFC anode and biocathode and proved to outperform the bare carbon paper [74,75].

Physical deposition to enable nanomaterial self-assembly on the planar surfaces is a convenient method to form 3D structures. Meanwhile the pore size of these modified 3D structures is usually less than 1 μm, which cannot facilitate biofilm formation in the electrode scaffold (electroactive bacteria have μm size). As a result, the BES performance with such an electrode is usually not competitive with 3D electrodes with open macroporous structure.

#### 2.2.2. In Situ Growth

**Figure 2:**
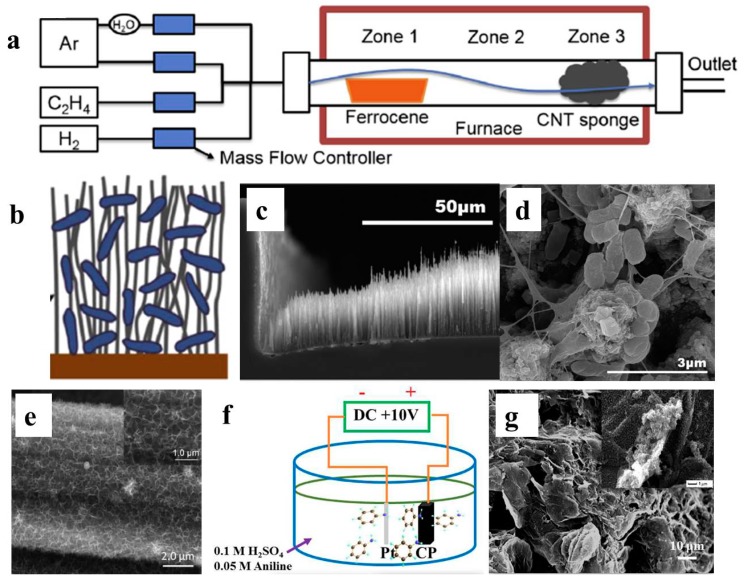
(**a**) Schematic of porous 3D CNT sponge fabrication by CVD [59]; (**b**) Schematic of biofilm attachment in CNT matrix with long and loose structure [78]; (**c**,**d**) SEM images of vertically aligned, forest like MWCNT deposited on silica wafer chamber via CVD [77]; (**e**) SEM image of porous MnO_2_ 3D frame in-situ growth on carbon paper via electrochemical reduction [79]; (**f**,**g**) Schematic and SEM image of in-situ fabricated graphene/PANI composite electrode on graphite paper surface [81]. Reproduced with permission from Elsevier, American Chemical Society and Wiley.

Compared with random assembly by physical deposition, 3D structures with well-aligned and symmetrical structures can be formed via an in situ growth strategy. Chemical vapor deposition (CVD) is the most frequently used technique for in situ growth of 3D carbon structures on planar surfaces [59,76,77,78]. Erbay et al. synthesized a 3D CNT sponge with high porosity and random tangles by using ferrocene as catalyst for CVD growth (Figure 2a). The fabricated CNT sponge was then applied as an MFC anode and the performance with different anode chamber sizes was compared. When the highest chamber/electrode volume ratio (400-fold) was adopted, power density normalized to electrode volume was as high as 2130 W/m^3^; meanwhile the value was only 283 W/m^3^ when the volume ratio was 1.7 [59]. By preparing different catalyst layers, five types of CNT with different morphology were synthesized via in situ CVD growth on stainless steel mesh (SSM). Performance comparisons confirmed that long and loosely-packed CNT without amorphous carbon was most suitable for BES applications [78]. The improved performance was due to the structure that is feasible for 3D biofilm formation among the CNT sponge (Figure 2b). Mink et al. fabricated a microliter-sized MFC (1.25 μL) and vertically aligned, forest-shaped MWCNT was synthesized as an anode via CVD. High power output (500 nW, equal to 396 W/m^3^ normalized to chamber volume) was achieved and was believed to capable of driving ultra-low power devices, like integrated nanobiosensors and the 29.6 pW Phoenix processor [77].

In addition to CVD, electrochemical deposition is another choice for in situ 3D structure preparation for BES. A conductive 3D polyaniline (PANI) nanowire network was successfully fabricated on an indium tin oxide (ITO) electrode via electrochemical polymerization. When applied as an MFC anode, a maximum power density of 2.3 W/m^2^ was achieved with mixed culture [58]. Gong et al. electrochemically deposited a porous MnO_2_ 3D framework on carbon paper via in situ reduction. MFC with a modified carbon paper anode achieved a maximum power density of 0.596 W/m^2^, which was almost nine times that of bare carbon paper. The improved performance was ascribed to enhanced pseudo-capacitance with the MnO_2_ 3D frame formation [79]. By employing a direct current voltage to the graphite paper electrode, graphene layers can be partially exfoliated and form 3D graphene structures on graphite paper electrodes. MFC with this graphene/GP electrode obtained a maximum power density of 2.36 W/m^2^ [80]. Furthermore, when aniline monomer was employed into the electrolysis system, a graphene/PANI composite structure was formed on the graphite paper electrode, and maximum power density of corresponding MFC was 4.44 W/m^2^. The graphene layer structure on electrode surface enables macroporous structure formation and partially explains the high performance achieved [81].

### 2.3. Monolithic 3D Electrode from 3D Porous Template

Although a 3D structure can form on flat 2D electrode via spread or in situ growth, the pore sizes of the corresponding electrodes were usually very small. Since the thickness of biofilm can be up to several tens of micrometers [120,121], the target for 3D biofilm formation throughout the electrode scaffold will require the corresponding electrode pore size to be no less than 100 μm. As a result, using a 3D porous template for modification and fabrication is the most successful strategy to prepare monolithic 3D electrodes.

#### 2.3.1. 3D Electrodes Fabricated from Conductive Porous Templates

**Figure 3:**
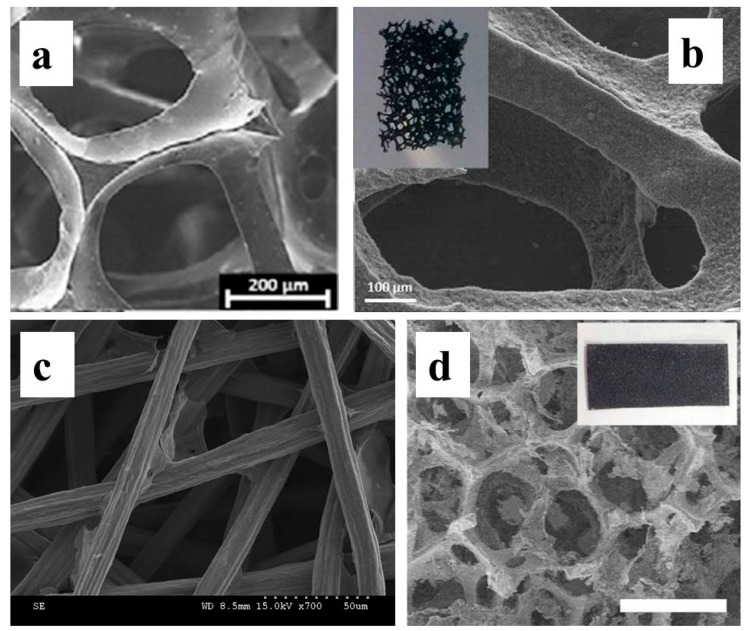
SEM image of (**a**) reticulated carbon foam [83]; (**b**) Carbon Nanoweb modified reticulated vitreous carbon [84]; (**c**) graphene oxide modified stainless steel fiber felt [85]; (**d**) rGO coated nickel foam, the bar is 200 μm [87]. Reproduced with permission from Elsevier and the Royal Society of Chemistry.

Modifying the commercial 3D conductive template is a promising choice. Reticulated vitreous carbon (RVC), a kind of conductive monolithic carbon material with open structure, is one of the most appealing candidates for BES application (Figure 3a). RVC has been used as an electrode for a long period of time [122]. However, the interest for employing RVC in BES arose recently. Lepage et al. made an early trial with commercially purchased RVC [83]. The used RVC had a large surface area (3750 m^2^/m^3^) and high porosity (95%) which was superior than the reported packed bed MFC [55,61]. The average strut and pore size of RVC were 100 and 320 μm, indicating the open structure was feasible for biofilm formation into the carbon matrix. Meanwhile the image of confocal laser microscopy (CLMS) was not provided, thus it cannot be determined whether the biofilm formed on the internal layers of RVC. No electrode modification was conducted and maximum power density of the corresponding MFC was 0.11 W/m^2^ (projected area), 1.72 W/m^3^ (anode chamber volume), and 39.6 W/m^3^ (electrode volume). This performance was not competitive to those modified plane electrodes or conventional packed bed MFC and brush electrodes [63]. However, rather high electricity output (68 A/m^2^, higher than all the previous reports when the work was published) was achieved in BES after the RVC was modified by a carbon nanoweb [78,84]. Biofilm cyclic voltammetry (CV) analyses at turnover conditions confirmed that the carbon nanostructure impressively improved the EET efficiency of BES.

Hou et al. used a stainless steel fiber felt (SSFF) as conductive porous support and modified it with activated carbon, CNT, and GO for MFC anode applications. The maximum power density was achieved with GO-modified SSFF as 2.14 W/m^2^ (projected area), 7.7 W/m^3^ (anode chamber volume), and 2140 W/m^3^ (electrode volume). The SSFF porosity (78%) and pore size (15.7 μm) was smaller than RVC [85]. However, a direct comparison between these two conductive templates was not conducted. As with previous works, the evidence for internal biofilm formation was not provided.

Nickel foam is another conductive porous support that has been widely used for various 3D electrode fabrications. Qiao et al., for the first time, utilized nickel foam as the template and decorated it with PANI/TiO_2_ nanostructured composite for BES application [86]. A maximum power density of 1.49 W/m^2^ was achieved in *E. coli*-inoculated MFC. Wang et al. modified the nickel form with rGO by autoclaving the nickel foam in GO solution, followed by a hydrogen reduction at 400 °C [87]. The average pore size estimated from SEM image was around 150 μm. The maximum power density of *Shewanella*-inoculated MFC with this 3D electrode was 0.663 W/m^2^ (anode projected area) or 663 W/m^3^ (electrode volume), which was 15–20 times that of unmodified nickel foam and other commercial electrodes. These results demonstrate that although the 3D nature of these conductive porous templates provides large surface area for biofilm formation, proper modification could impressively improve the BES performance. As nanomaterial modification mainly changes the nanostructure of the 3D surface and the open macroporous structure remains, a synergistic effect of 3D surface and modification can be anticipated when they are combined.

#### 2.3.2. 3D Electrodes Fabricated from Non-Conductive Porous Template

The MFC performance with modified conductive 3D electrodes was usually high compared with plane electrode. Meanwhile, there are only a few choices for commercial 3D conductive porous template. Surface modification of a non-conductive 3D porous template with conductive nanomaterials is, thus, potentially applicable since it would provide an extra choice for the 3D porous template and enables fabrication of a 3D electrode with various structures. The main concern is the reduced electrode conductivity due to the use of a non-conductive template.

**Figure 4:**
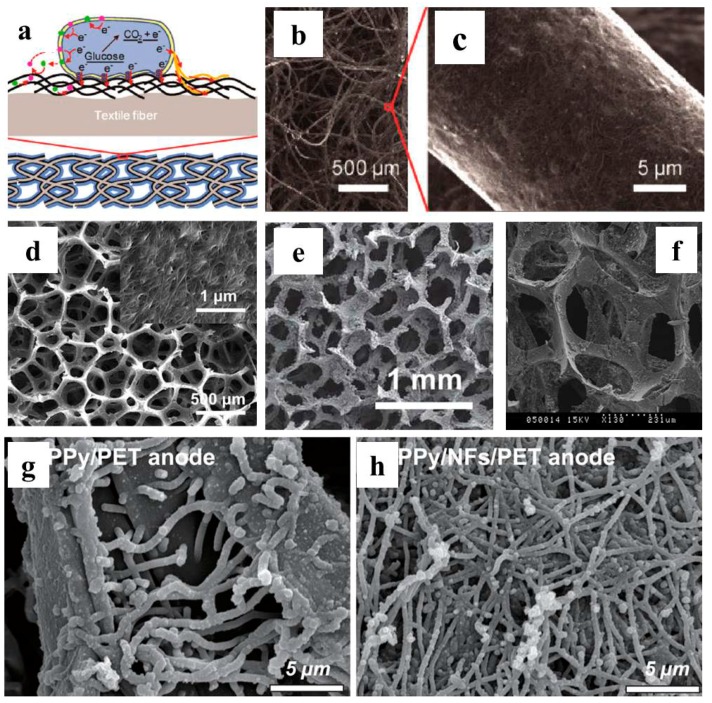
(**a**) Schematic of 3D CNT-textile electrode; SEM image of (**b**,**c**) textile fiber coated with CNT [88]; (**d**) CNT coated sponge [89]; (**e**) graphene coated sponge [90]; (**f**) nickel coated sponge [91]; (**g**,**h**) PPy coated PET and Nano fiber PET textile [123]. Reproduced with permission from American Chemical Society, the Royal Society of Chemistry and Elsevier.

Xie et al. reported a successful attempt by coating the textile with CNT to fabricate a conductive 3D CNT-textile anode (Figure 4a–c) [88]. The coating was accomplished by repeatedly dipping and drying the textile in a SWCNT-containing solution. The as-prepared 3D electrode exhibited a good conductance of around 50 S/cm. When applied as an MFC anode, the maximum power density was 1.1 W/m^2^. The charge transfer resistance was greatly reduced, which was ascribed to the efficient electron transfer between CNT and electroactive biofilm. Electrode and biofilm cross-section SEM images confirmed that the biofilm was restricted to the outer surface of the textile, indicating the porosity and pore size of the as-prepared electrode needs further improvement. Non-conductive sponge was further used, substitutive of the textile as the 3D template. A similar dip-drying process was conducted to fabricate a 3D CNT-coated sponge electrode. This CNT-sponge exhibited lower internal resistance, more tunable and uniform macrostructures compared with CNT-textile (Figure 4d). The average pore size of 500 μm enabled biofilm perpendicular formation along the 3D scaffold. When employed as MFC anode, the maximum power density increased to 1.99 W/m^2^, which is 80% higher than that with CNT-coated textile [89]. Furthermore, graphene was also successfully adopted for sponge coating to fabricate a 3D electrode with similar preparation method (Figure 4e) [90]. Pieces of stainless steel mesh were pasted to the insides the graphene sponge as the electron collector. The stainless steel mesh increased the electrode conductivity and MFC with graphene-sponge electrode achieved a maximum power density of 1.57 W/m^2^. Liu et al. coated the sponge with nickel via CVD (Figure 4f) [91]. The maximum power density of this MFC with the nickel-sponge electrode was 0.993 W/m^2^.

Tao et al. fabricated a 3D template via spray coating of PVA-co-PE solution onto a pre-treated PET surface (Figure 4g,h) [123]. Then conductive polypyrrole was in situ chemically polymerized with anthraquinone-2-sulfonic acid sodium (AQDS, electron shuttle) as dope. This 3D electrode was further used as an *E. coli*-inoculated MFC anode and the maximum power density was 2.42 W/m^2^.

Modifying a 3D porous template with proper nanomaterial is an effective strategy to fabricate 3D electrodes. Good conductivity and proper pore size are two prerequisites for the BES application. Various successful porous templates have been made. Meanwhile, only a few proved their value. Rational design of new porous template probably stands for the future of 3D electrode modification.

#### 2.3.3. 3D Electrodes Fabricated with a Sacrificial Porous Template

Direct, free-standing 3D electrode fabrication from sacrificial templates is another strategy to prepare a 3D electrode with an open macrostructure. The attempts of preparing 3D electrodes from sacrificial templates can be classified into two groups. The first method is coating the 3D porous template with conducitive materials and then removing (e.g., reflux in acid) the template to obtain the free-standing monolithic 3D electrode. Compared with modifying porous supports, an extra template removal procedure was employed. The 3D electrode can also be prepared by carbonizing (under the protection of inert gas) proper carbon-containing 3D porous templates. Conductive monolithic 3D electrodes can form after hydrogen, oxygen, and other vaporable elements were removed by the carbonization process. Compared with modifying and coating strategies, removing the template not only increases the electrode porosity, more importantly, it broadens the choice of 3D porous templates.

**Figure 5:**
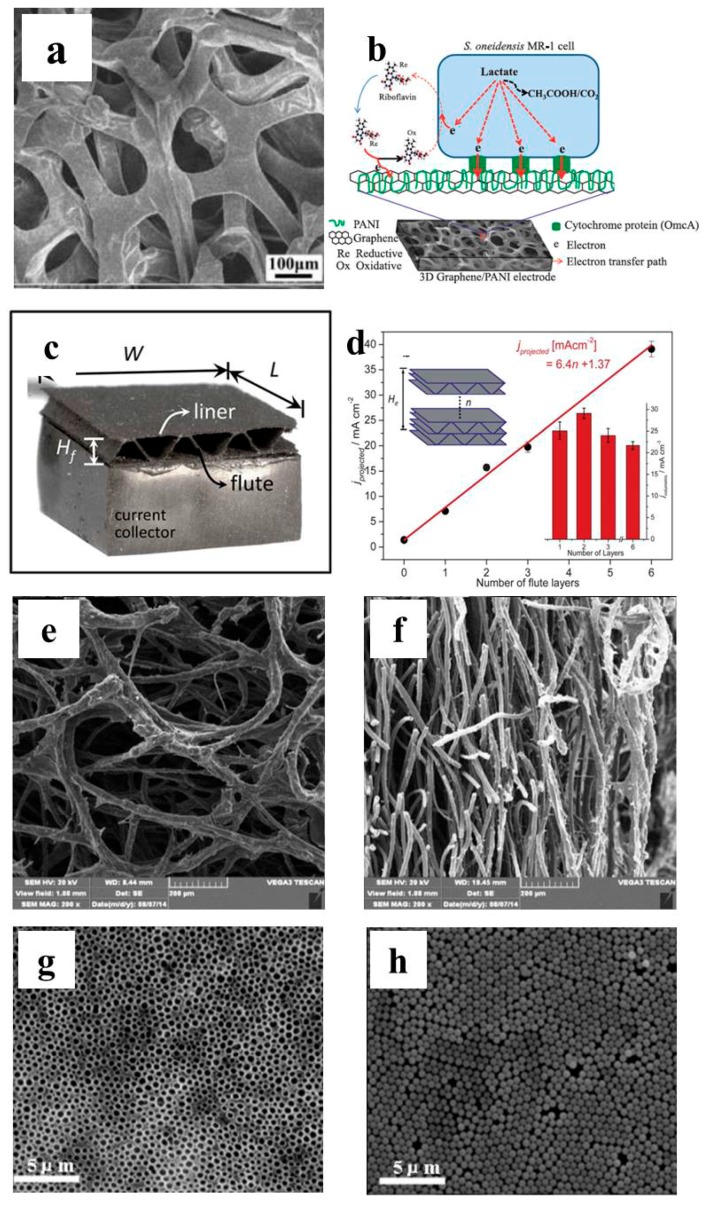
(**a**) SEM image of macroporous structured graphene/PANI monolithic electrode fabricated from in-situ CVD graphene synthesis on nickel foam template and followed by PANI chemical polymerization; (**b**) Schematic illustrating the interface electron transfer between *S. oneidensis* MR-1 and graphene/PANI electrode [92]; (**c**) Photographic image of single layered corrugated carbon electrode; (**d**) Dependence of BES steady state current density on the number of layered corrugated carbon electrode [93]; (**e**,**f**) front and side-surface SEM image of carbonized towel electrode [94]; (**g**,**h**) SEM image of SiO_2_ template and porous carbon with defined pore size [95]. Reproduced with permission from American Chemical Society, the Royal Society of Chemistry and Elsevier.

Yong et al. first reported such a macroporous and monolithic electrodes for BES 103. A graphene layer was first deposited on nickel foam via CVD. The nickel foam template was then etched away by refluxing in 3M HCl, enabling the formation of 3D free-standing graphene foam. Then this graphene foam was chemically modified with PANI to increase the hydrophilicity and conductivity (Figure 5a). The monolithic electrode was then used as a *Shewanella*-inoculated MFC anode and a maximum power density of 0.77 W/m^2^ (projected area) and 768 W/m^3^ (electrode volume) was achieved, higher than previously reported *Shewanella* MFCs. SEM imaging confirmed the formation of internal biofilm, verifying the importance of a macroporous 3D structure. Meanwhile electrochemical analysis demonstrated significantly improved *Shewanella* EET. Chen *at al.* prepared single-wall layered corrugated carbon (LCC) electrodes through carbonization of recycled paper with one flute layer as the template 104. The multi-layer LCC electrode can be prepared by simply pasting the single wall template with corn starch glue. These LCC electrodes were then applied for BES characterization via a constant potential discharge. The influence of LCC geometry size was investigated. The most impressive result obtained from this work is that the steady state current output increased linearly with number of LCC layers, providing solid support on the role of 3D structure for performance improvement (Figure 5d). A maximum current density of 390 A/m^2^ (six layers) was achieved in this work and was the highest of the reported works. Wang et al. fabricated a carbonized towel electrode with freely standing and twisted fibers from commercial towel (Figure 5e,f) [94]. A maximum current density of 8 A/m^2^ was achieved in a BES test. The successful use of commercial towel broadens the choice of 3D templates and we may expect more inspired reports in the future.

In addition to using commercial templates for 3D electrode preparation, Chen et al. developed a strategy for porous carbon electrode fabrication with SiO_2_ templates [95]. The chemically-synthesized SiO_2_ template was mixed with sucrose in H_2_SO_4_ solution. The mixture was then carbonized and the porous structure was obtained by etching the SiO_2_ template with 10% HF solution. The maximum power density of MFC with this porous carbon electrode was 1.6 W/m^2^ (*E. coli* inoculum), which was almost four times that of carbon felt. Liu et al. further modified the porous carbon with TiO_2_ nanoparticles and the MFC equipped with this modified porous carbon electrode achieved a maximum power density of 0.973 W/m^2^ [96]. Etching the template for the porous structure is potentially a valuable strategy to fabricate macroporous 3D electrodes. However, the average pore size through SiO_2_-etching was only 400 nm in those works. 3D electrode fabrication that could facilitate internal biofilm formation requires the use of a template with a size larger than 100 μm in case the porous structure is expected to develop from template etching.

**Figure 6:**
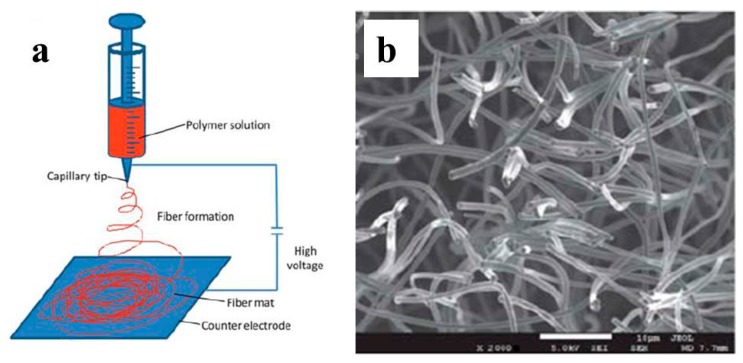
(**a**) Schematic of electrospunning setup; (**b**) SEM image of 3D porous carbon fiber mats fabricated by electrospun, the bar is 10 μm [97]. Reproduced with permission from the Royal Society of Chemistry.

Electrospinning is a powerful tool for preparing 3D templates. Chen et al. conducted the pioneering work using electrospinning to fabricate 3D carbon fiber mats (Figure 6) [97]. Carbon black was added to polyacrylnitrile and resulted in a loose 3D structure with high porosity. Three ways for carbon fiber mat preparation were investigated include gas-assisted electrospinning, electrospinning, and solution blown methods. The as-prepared carbon fiber mats were carbonized to obtain free standing 3D porous electrodes. These 3D electrodes were used as BES anode. The maximum current density of 30 A/m^2^ was achieved with the electrode by gas-assisted electrospinning.

**Figure 7:**
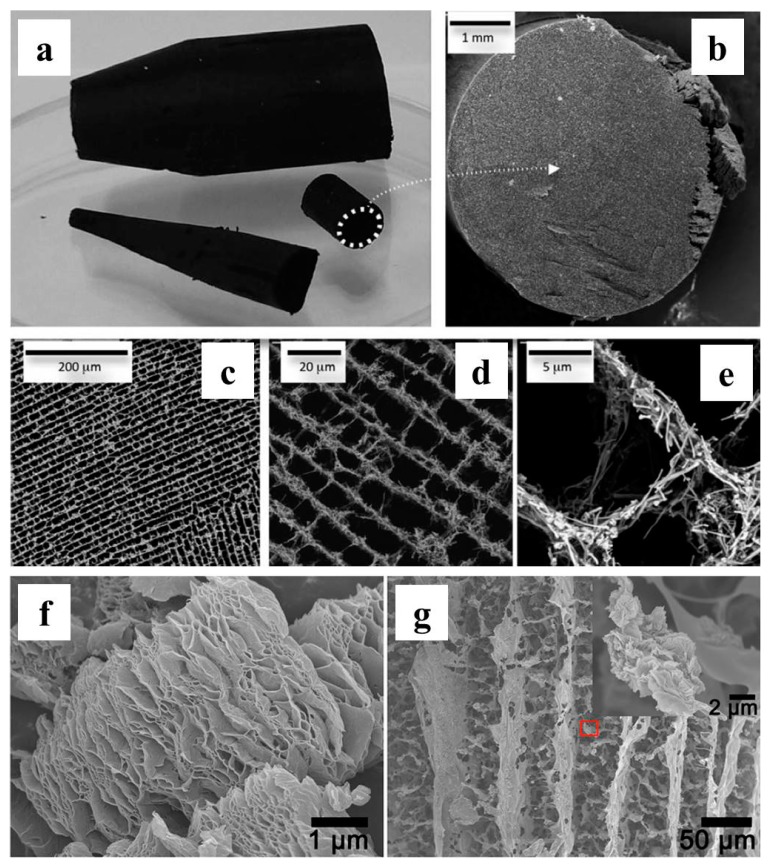
(**a**,**b**) Photograph and (**c**–**e**) SEM images of chitosan/MWCNT 3D scaffold prepared by with ice template [99]; (**f**,**g**) SEM images of chitosan/vacuum stripped graphene scaffold [100]. Reproduced with permission from the Royal Society of Chemistry, Elsevier and American Chemical Society.

Compared with chemically-synthesized templates, ice segregation-induced self-assembly (ISISA) can provide a much larger “ice template” for macroporous 3D electrode fabrication. Katuri et al. prepared a chitosan/MWCNT 3D scaffold through ISISA (Figure 7a–e) [99]. Generally, a mixture solution containing functionalized MWCNT and chitosan was dripped (at a rate of 2.7 mm/min) into a cold bath at −196 °C and ambient pressure. The unidirectional frozen sample was then freeze-dried, resulting a highly-conductive monolithic electrode. The porosity of the monolithic electrode can be tuned by adjusting the dripping rate and the as-prepared chitosan/MWCNT 3D scaffold electrode exhibited an average pore size around 10 μm. This electrode was then employed as the anode for *Geobacter sulfurreducens*-inoculated BES. The maximum power density achieved in MFC was 2.87 W/m^2^ (electrode area) and 2000 W/m^3^ (electrode volume), both of which were among the highest. He et al. further fabricated a chitosan/vacuum stripped graphene (VSG) 3D scaffold electrode via ISISA. The chitosan/VSG scaffold exhibited a well-aligned layered structure with layer spacing around 30–50 μm, which effectively facilitated interior biofilm formation (Figure 7f,g). When applied as the anode of a *Pseudomonas aeruginosa*-inoculated MFC, the maximum power density improved to 78 times that of carbon cloth. Chen et al. prepared a similar 3D graphene sponge using an ice template [101]. A comparison study on the cooling rate and sponge morphology was conducted. The results indicated that slowly cooling resulted in a larger ice crystal size and finally enabled the formation of graphene sponge with a macroporous structure. The maximum power density of the MFC with this graphene sponge was 0.71 W/m^2^.

#### 2.3.4. 3D Electrodes Fabricated from Natural Porous Template

The development of 3D electrodes via template fabrication can be, somehow, viewed as a history of exploring the valuable template. In addition to those commercially or lab prepared templates, versatile natural products also inspired researchers as they naturally occupy 3D structures.

**Figure 8:**
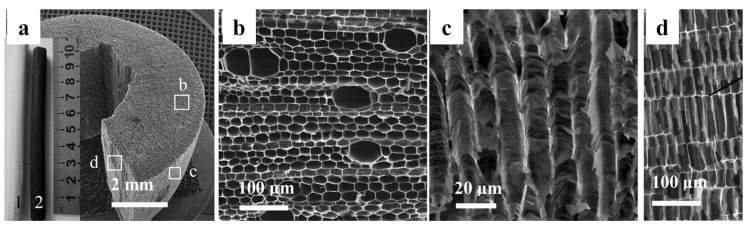
Images of 3D-KSC. (**a**) Overview image of a piece of cleaved 3D-KSC. The insets are photographs of kenaf stalk before (1) and after (2) carbonization; (**b**) Vertical sectioned SEM image magnified from position b; (**c**) SEM Image magnified from position c; (**d**) Longitudinal sectioned SEM image magnified from position d [102]. Reproduced with permission from Wiley.

Chen et al. made a pioneering attempt by carbonized kenaf (a kind of crop plant possessing an ordered three-dimensional macroporous architecture) to prepare a 3D macroporous carbon electrode (3D-KSC) with good conductivity for MFC anodes (Figure 8) [102]. SEM images confirmed that thick biofilms formed both on the outer and inner surface of KSC electrode. The maximum current density of BES with this 3D KSC electrode was 32.5 A/m^2^, which was almost three times that of graphite rod. They further fabricated an RVC electrode through pomelo peel carbonization [103]. MFC testing confirmed that this natural product-derived RSC electrode outperformed the commercial RVC electrode. The influence of the as-prepared electrode thickness on performance was investigated. The performance comparison revealed that although current density normalized to projected area increased along with electrode thickness, the electrode volume normalized current density decreased by 55% when the thickness increased from 0.76 mm to 5.78 mm. These results indicate that biofilm could not uniformly form from the external to internal 3D scaffold, although the average pore size estimated from SEM image was as large as 200 μm.

Yuan et al. prepared a nanostructured macroporous 3D electrode via loofah sponge carbonization [104]. The loofah sponge carbon (LSC) was then modified by in situ PANI polymerization to form a PANI/LSC electrode. When employed as an MFC anode, this PANI/LSC outperformed unmodified LSC, commercial RVC, graphite plate, carbon felt, and also graphene-coated sponge electrodes. Detailed electrochemical analyses revealed a significantly improved EET efficiency. This LSC electrode was further modified by TiO_2_ core-shell nanoparticles to improve the pseudo-capacitance of the LSC electrode [105]. The electrochemical analyses confirmed a positive correlation between specific capacitance and MFC power density. Karthikeyan et al. also fabricated porous carbon electrode by carbonization of three kind of plants (soft king mushroom, hard wild mushroom, and corn stem) [106]. Bacteria-electrode interfacial electron transfer was detailed compared within individual electrodes. The improved MFC performance was explained due to the enhanced biofilm electroactivity and improved heterogeneous electron transfer rate.

### 2.4. 3D Bioelectrode with Hybridized Biofilm

In most BES, regardless the various electrode material and configuration used for their specific purposes, the electroactive biofilm are supposed to gradually evolved after BES set up, meaning the electrode fabrication and biofilm formation are temporal separated. The formation of mature electroactive biofilm is time cost and several days to months are required, depended on inoculum and BES operation conditions [124]. The substitutive strategy is encapsulating a defined population of cells with proper material to shape engineered biofilms which preserve the integrity of whole cells with a defined physiological status, or developing a bacteria/material hybridized bioelectrode through self-assembly, in which electrode fabrication and biofilm formation are spontaneously achieved [109,115].

#### 2.4.1. Bacteria Immobilization

**Figure 9:**
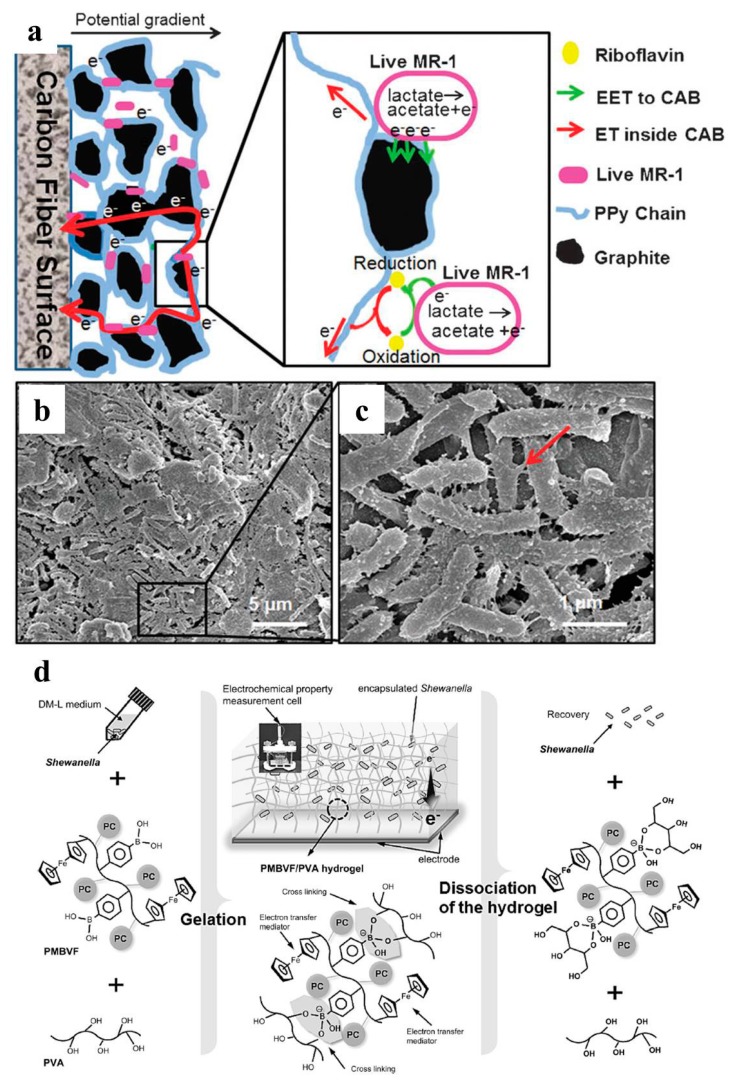

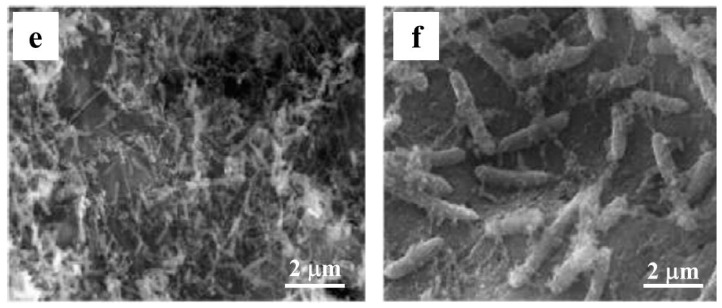
(**a**) Schematic and (**b**,**c**) SEM image of artificial conductive biofilm. *Shewanella* cells were immobilized in a conductive matrix consisted of graphite particle and in-situ synthesized PPy [109]; (**d**) Schematic of PMBVF/PVA hydrogel formation mechanism and design of hydrogel/bacteria hybrid biofilm [110]; (**e**,**f**) SEM image of *Shewanella* cells immobilized to PHBV/CF composites via silica vapor deposition [111]. Reproduced with permission from the Royal Society of Chemistry, Elsevier and American Chemical Society.

Yuan et al. reported a successful trial by artificial immobilizing electroactive mixed culture with carbon nanoparticles [108]. The electroactive bacteria were first mixed with carbon nanoparticles (ca. 300 nm) and Teflon emulsion to form a carbon paste. The paste was then directly spread on the carbon cloth to achieved direct electroactive bacteria immobilization. The maximum power density of the MFC with the immobilized bacteria culture was 1.94 W/m^2^. Yu et al. developed a layer by layer in situ polymerization strategy to immobilize the *Shewanella* cells into a graphite particle and PPy matrix (Figure 9a–c) [109]. This bacteria-immobilized artificial biofilm exhibited good conductivity (3.2 mS/cm), high bacteria viability and stable electroactivity when adopted as MFC anode (no electroactivity decay after 600 h discharge). The maximum power density of MFC was 0.207 W/m^2^, almost 18 times of carbon cloth. Lin et al. developed a type of biocompatible hydrogel to encapsulating *Shewanella* [110]. The bacteria-encapsulated hydrogel was prepared by blending the *Shewanella*-contained PMBVF medium with PVA medium. This bacteria hydrogel showed good bacteria viability even after long-term storage. The maximum current density of BES with this *Shewanella* encapsulated hydrogel electrode was 0.082 A/m^2^. Luckarift et al. developed another strategy for *Shewanella* immobilization (Figure 9e,f) [111]. A PHBV/CF scaffold was first prepared by press molding and water dissolution to remove a sucrose template. *Shewanella* cells were then immobilized via vapor hydrolysis of the silica hydrogel. The maximum current density of BES with this *Shewanella* hydrogel was 0.072 A/m^2^. Both of the *Shewanella*-encapsulated hydrogels exhibited low electroactivity, which may be ascribed to the limited hydrogel conductivity.

#### 2.4.2. Self-Assembled Hybrid Biofilm

Although direct electroactive bacteria immobilization is an effective strategy for fast biofilm formation, the performance of BES with most artificial biofilms is usually low, which is probably due to the low conductivity and porosity. Instead, self-assembled hybrid biofilms (SAHB) usually exhibited excellent electroactivity since the formation of such hybrid biofilms are “electroactive bacteria selected” rather than “artificially selected”. The study of SAHB is still in the initial stage. To form a SAHB, certain nanomaterials (for example graphene oxide) were supplied into the electrolyte along with bacteria inoculum. During the electroactive biofilm formation, solution-dispersed nanomaterials were attached to bacteria due to the physical and chemical interaction and, as a result, a bacteria/nanomaterial hybrid formed on the electrode surface. The above process was repeated and finally a hybrid biofilm was assembled on the surface of the electrode. Due to the high conductivity of the hybrid biofilm, the SAHB can develop to a thickness of centimeters, which is a hundred-fold of naturally-formed biofilm on a plane surface.

**Figure 10:**
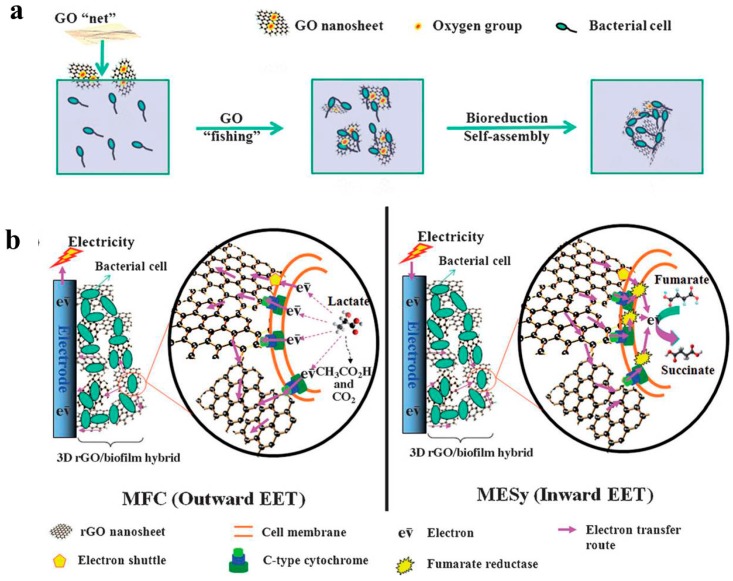
Schematic of (**a**) self-assembly of the 3D macroporous rGO/bacteria hybrid biofilm by a fishing process; (**b**) the proposed mechanism of bidirectional EET [115]. Reproduced with permission from Wiley.

Nakamura et al. added α-Fe_2_O_3_ into *Shewanella*-inoculated BES, and a light-induced α-Fe_2_O_3_/bacteria hybrid network was then self-assembled on ITO surface and the electroactivity was increased 300-fold [112]. The electron transfer mechanism along the α-Fe_2_O_3_/bacteria networks was proposed. Park et al. reported a SAHB with Fe_3_O_4_/CNT nanocomposite as absorptive material and *E. coli* as electroactive bacteria [113]. The formation of SAHB was achieved in a shaking incubator and increased the maximum power density of MFC to 0.83 W/m^2^. Furthermore, Yong et al. reported a self-assembled 3D reduced graphene oxide (rGO)-hybrid biofilm with *Shewanella oneidensis* MR-1 as the electroactive bacteria [115]. A detailed mechanism for SAHB biofilm formation was proposed for the first time (Figure 10). Briefly speaking, non-conductive GO nanosheets captured bacteria by a “fishing mode” and were then reduced to conductive rGO, generating a 3D hybrid biofilm with a macroporous interconnected structure. The maximum power density of the 3D biofilm was around 22 times higher than that of natural biofilm. Another interesting result of this work is that the SAHB both significantly enhanced the outwards (from bacteria to electrodes, 22 times), and inwards (from electrodes to bacteria, 74 times), EET.

## 3. Challenges and Perspectives

Versatile strategies have been developed for 3D electrode fabrication in BES studies. Generally, the 3D electrode suitable for BES must have good conductivity, large surface area, and suitable surface properties for bacteria attachment and electron transfer. The most important criterion for a 3D electrode is the open macroporous structure that can facilitate both external and internal biofilm formation.

Nanomaterial self-assembly can help to develop a 3D nanostructure on a 2D electrode, which could facilitate the EET between the bacteria and electrode interface. Meanwhile the pore size is usually small and cannot facilitate the interior biofilm growth. Compared with conventional and 2D electrode modification, modifying and coating a 3D macroporous template is promising for preparing a monolithic 3D electrode. The main problem is the limited choice of proper commercial templates. The 3D porous template could also be removed or post-treated during electrode preparation, which can reduce the electrode weight and increase the choice for available templates. 

One of the future developments in 3D electrodes in BES is exploring and designing proper 3D porous templates. Impressive achievements in designing 3D templates can be anticipated with the developments of material science and nanotechnology. Nature will continuously motivate researchers to explore their products as proper 3D templates. Meanwhile, inspiration for biomimetic template synthesis may arise in the future since many living organism are born artists for architecting 3D buildings. Fundamental study on searching for the optimal conditions for 3D electrodes is another aspect for the future. Modelling studies for simulating the behavior of electroactive biofilm in the 3D electrode need to be conducted.

In addition to the 3D electrode design, bacteria immobilization and hybrid biofilm are another group of strategies to form a 3D bioelectrode. Bacteria immobilization can greatly reduce the time consumption for biofilm formation. More studies on immobilization strategy and application perspectives exploration areas, like biosensors, are required. SAHB may be the most interesting and promising strategy for 3D electrode development. The conductive hybrid biofilm can be rational manipulated. Meanwhile, the relative research is at initial stage. The underlying mechanism for biofilm/material self-assembly needs to be elucidated. Further development shall extend the materials and electroactive bacteria applicable for developing SAHB, and enable more versatile applications, like pollution removal and biofuel production. Strategies for tuning the SAHB interior structure and properties need to be explored.

Along with the rapid development in the preparation of 3D electrodes for BES applications, there is an increasing necessity to establish standard criteria for 3D electrode characterization and application. For example, there should be clear definition and criteria that are adopted to characterize the 3D electrode, including porosity and biofilm formation status. When applied in BES, there should also be standard criteria for data acquisition and analyses. The electrochemical analytical routine used to characterize and compare the electrochemical activity of biofilm on corresponding 3D electrodes needs to be established. 

## Figures and Tables

**Table 1:** Representative 3D electrodes in BES.

BES Type ^#^	Inoculum	3-D Strategy ^$^	Electrode Configuration ^&^	j^a^ (A/m^2^)	P^a^ (W/m^2^)	P^b^ (W/m^3^)	P^c^ (W/m^3^)	Reference
S-MFC/air cathode	MFC effluent	1	Graphite granules	*	0.6	48	102.1	[55]
D-MFC/ferricyanide	Anaerobic sludge	1	Granule activated carbon	*	*	11.9	20.2	[57]
D-MFC/ferricyanide	MFC effluent	1	Graphite granules	*	*	257	*	[60]
S-MFC/Pt-air cathode	Wastewater	1	Granule activated carbon	*	0.245	7.2	*	[61]
S-MFC/Pt-air cathode	Anaerobic sludge	1	Irregular graphite granules	1.5	0.08	2.0	2.7	[62]
D-MFC/biocathode	MFC effluent	1	Granule activated carbon	0.91	0.194	9.72	16.2	[63]
S-MFC/Pt-air cathode	MFC effluent	1	Granule activated carbon	*	0.813	81.2	*	[64]
D-MFC/biocathode	MDC effluent	1	Granule activated carbon	4.44	1.05	21.2	36.0	[65]
S-MFC/Pt-air cathode	MFC effluent	1	Treated carbon brush	8.4	1.37	34.7	71.4	[50]
S-MFC/Pt-air cathode	MFC effluent	1	Carbon brush	10	1.24	24.9	42.1	[66]
D-MFC/ferricyanide	MFC effluent	1	Carbon brush	9.45	2.1	210	373	[67]
D-MFC/ferricyanide	Anaerobic sludge	2	rGO/PANI modified CC	3.4	1.39	11.2	*	[68]
D-MFC/ferricyanide	*Escherichia coli*	2	rGO/SnO_2_ modified CC	3.4	1.62	*	*	[69]
Marine benthic MFC	*	2	MWCNTs/MnO_2_ modified GP	0.75	0.11	*	*	[70]
D-MFC/ferricyanide	*Escherichia coli*	2	MWCNTs/Pt NP modified CP	*	2.45	*	*	[71]
D-MFC/ferricyanide	*Escherichia coli*	2	MWCNTs/SnO_2_ coated GCE	3.5	1.421	*	*	[72]
D-MFC/ferricyanide	Anaerobic sludge	2	PEI/graphene modified CP	1.7	0.368	3.9	*	[73]
D-MFC/biocathode	Anaerobic sludge	2	CNT/chitosan modified CP	1.6	0.189	*	*	[74]
S-MFC/Pt-air cathode	Anaerobic sludge	2	CNT/chitosan modified CP	0.8	0.132	*	*	[75]
S-MFC/Pt-air cathode	Anaerobic digester	2	CNT in-situ growth on SSM	*	1.87	8.5	*	[76]
D-MFC/ferricyanide	Wastewater	2	CNT in-situ growth	0.197	0.0196	396	*	[77]
D-MFC/ferricyanide	Wastewater	2	CNT sponge	8	2.82	14.1	943	[59]
D-MFC/ferricyanide	Anaerobic sludge	2	CNT in-situ growth on SSM	6.5	3.36	6.72	*	[78]
D-MFC/air	Active sludge	2	CP modified with MnO_2_	3	0.596	14.9	*	[79]
S-MFC/Pt-air cathode	MFC anode effluent	2	Graphene modified GP	9.45	2.36	16.5	472	[80]
D-MFC/ferricyanide	Active sludge	2	Graphene/PANI modified GP	10.5	4.44	29.6	2220	[81]
D-MFC/ferricyanide	*Shewanella oneidensis*	3	PANI/CNT modified CF	1.9	0.257	1.32	*	[82]
D-MFC/air	MFC effluent	3	Commercial RVC	1.04	0.11	1.72	39.4	[83]
Half-cell MFC, +0 V	MFC effluent	3	CNT modified RVC	68	*	*	*	[84]
D-MFC/ferricyanide	MFC effluent	3	CNT graphene modified SSM	8.1	2.14	7.7	2140	[85]
D-MFC/ferricyanide	*Escherichia coli*	3	PANI/TiO_2_ coated nickel foam	8	1.49	0.99	*	[86]
D-MFC/ferricyanide	*Shewanella oneidensis*	3	Nickel foam coated with rGO	3	0.663	27	663	[87]
D-MFC/Pt-air	Domestic wastewater	3	Textile coated with CNT	7.2	1.1	0.599	*	[88]
D-MFC/Pt-air	Domestic wastewater	3	Sponge coated with CNT	21.3	1.99	1.32	995	[89]
D-MFC	MFC effluent	3	Graphene coated sponge	10.7	1.57	*	394	[90]
D-MFC/ferricyanide	Wastewater	3	Nickel coated sponge	4.3	0.993	5.53	*	[91]
D-MFC/ferricyanide	*Shewanella oneidensis*	3	Monolithic graphene electrode	4.5	0.77	0.512	768	[92]
Half-cell MFC, +0.2 V	MFC effluent	3	Layered corrugated carbon	390	*	*	*	[93]
Half-cell MFC, +0.3 V	Anaerobic sludge	3	Towel carbonization	8	*	*	*	[94]
S-MFC/Pt-air cathode	*Escherichia coli*	3	Porous carbon	13.5	1.6	14.5	*	[95]
S-MFC/Pt-air cathode	Anaerobic sludge	3	TiO_2_ modified porous carbon	3.69	0.973	48.6	*	[96]
Half-cell MFC, +0.2 V	MFC effluent	3	Carbonized polymer matrix	30	*	*	*	[97]
Half-cell MFC, +0.2 V	MFC effluent	3	Carbonized polymer matrix	20	*	*	*	[98]
D-MFC/air	*Geobacter sulfurreducens*	3	Chitosan/CNT scaffold	19	2.87	2.23	2000	[99]
D-MFC/ferricyanide	*Pseudomonas. aeruginosa*	3	Chitosan/graphene scaffold	5.25	1.53	*	*	[100]
D-MFC/ferricyanide	Anaerobic sludge	3	Graphene sponge	*	0.71	*	427	[101]
Half-cell MFC, +0.2 V	MFC effluent	3	Corp plant carbonization	32.5	*	*	*	[102]
Half-cell MFC, +0.2 V	MFC effluent	3	Pomelo peel carbonization	51.9	*	*	*	[103]
S-MFC/Pt-air cathode	MFC effluent	3	PANI modified LSC	12.4	2.54	27.2	509	[104]
S-MFC/Pt-air cathode	MFC effluent	3	TiO_2_ modified LSC	15	2.59	27.7	518	[105]
Half-cell MEC, +0.2 V	Anaerobic digester	3	Carbonized plant	31.2	*	*	*	[106]
S-MFC/denitrification	*Ochrobactrum anthropi*	4	Bacteria/copper powder	*	*	*	*	[107]
S-MFC/Pt-air cathode	Mixed culture	4	Bacteria/CNP paste	9.2	1.94	*	*	[108]
D-MFC/ferricyanide	*Shewanella oneidensis*	4	Bacteria/graphite/PPy matrix	0.8	0.207	*	*	[109]
Half-cell MFC, +0.2 V	*Shewanella oneidensis*	4	PMBVF/PVA/bacteria hydrogel	0.082	*	*	*	[110]
Half-cell MFC, −0.15 V	*Shewanella oneidensis*	4	EAB on polymer/GF scaffold	0.072	*	0.17	4.38	[111]
Half-cell MFC, +0.2 V	*Shewanella oneidensis*	4	Fe_2_O_3_/bacteria hybrid biofilm	0.23	*	*	*	[112]
D-MFC/ferricyanide	*Escherichia coli*	4	Fe_3_O_4_/CNT/bacteria hybrid biofilm	1.9	0.83	*	*	[113]
S-MFC/Pt-air cathode	Anaerobic sludge	4	rGO/bacteria hybrid biofilm	8.9	1.9	47.7	*	[114]
D-MFC/ferricyanide	*Shewanella oneidensis*	4	rGO/bacteria hybrid biofilm	5.2	0.843	*	*	[115]

^**#**^ S-MFC: single-chamber MFC, with air cathode; D-MFC: dual-chamber MFC, with liquid cathode; Half-cell MFC: MFC operated in single chamber at constant potential, (potential is referred to saturated Ag/AgCl electrode (+0.198 V vs. SHE) in the Table 1). ^$^ 1: conventional 3D electrode (packed bed or brush electrode); 2: 3D matrix fabricated on a 2D electrode; 3: monolithic 3D electrode from a 3D template; 4: 3D bioelectrode with hybridized biofilm. ^&^ rGO: reduced graphene oxide; PANI: polyaniline; MWCNT: multi-walled carbon nanotube; NP: nanoparticles; PEI: Polyethyleneimine; CNT: carbon nanotube; SSM: stainless steel mesh; PPy: polypyrrole; LSC: loofah sponge carbon; CNP: carbon nanoparticle; PMBVF/PVA: poly(2-methacryloyloxyethyl phosphorylcholine-co-*n*-butyl methacrylate-co-*p*-vinylphenylboronic acid-co-vinylferrocene)/poly(vinyl alcohol); EAB: electroactive bacteria. j^a^: maximum current density normalized to projected area; For packed bed MFC, j^a^ was calculated based on the separator area; For brush electrodes with a cylinder anode chamber, j^a^ was calculated based on cathode projected area. p^a^: power density normalized to projected area; For packed bed MFC, P^a^ was calculated based on the separator area; For brush electrodes with a cylinder anode chamber, P^a^ was calculated based on cathode projected area; P^b^: power density normalized to electrode chamber volume; P^c^: power density normalized to electrode volume. * Not provided and nor can be calculated with published data.
